# Spatial Variation and Determinants of Inadequate Minimum Meal Frequency among Children Aged 6–23 Months in Ethiopia: Spatial and multilevel analysis using Ethiopian Mini Demographic and Health Survey (EMDHS) 2019

**DOI:** 10.5334/aogh.4448

**Published:** 2024-06-25

**Authors:** Berhan Tekeba, Almaz Tefera Gonete, Melkamu Tilahun Dessie, Alebachew Ferede Zegeye, Tadesse Tarik Tamir

**Affiliations:** 1 Department of Pediatrics and Child Health Nursing, School of Nursing, College of Medicine and Health Sciences, University of Gondar, Gondar, Ethiopia; 2 Department of Medical Nursing, School of Nursing, College of Medicine and Health Sciences, University of Gondar, Gondar, Ethiopia

**Keywords:** Meal frequency, inadequate meal frequency, spatial analysis, multilevel analysis, young children, Ethiopia

## Abstract

*Introduction:* Minimum meal frequency is the number of times children eat in a day. Without adequate meal frequency, infants and young children are prone to malnutrition. There is little information on the spatial distribution and determinants of inadequate meal frequency at the national level. Therefore, we aimed to investigate the spatial distribution and determinants of inadequate meal frequency among young children in Ethiopia.

*Methods:* The most recent Ethiopian demographic and health survey data was used. The analysis was conducted using a weighted sample of 1,610 children aged 6–23 months old. The Global Moran’s I was estimated to assess the regional variation in minimum meal frequency. Further, a multivariable multilevel logistic regression model was fitted to identify factors associated with inadequate meal frequency. The AOR (adjusted odds ratio) at 95% CI (confidence interval) was computed to assess the strength and significance of the relationship between explanatory variables and the outcome variable. Factors with a p-value of <0.05 are declared statistically significant**.**

*Results:* This study revealed that the prevalence of inadequate meal frequency was found to be 30.56% (95% CI: 28.33–32.88). We identified statistically significant clusters of high inadequate meal frequency, notably observed in Somalia, northern Amhara, the eastern part of southern nations and nationalities, and the southwestern Oromia regions. Child age, antenatal care (ANC) visit, marital status, and community level illiteracy were significant factors that were associated with inadequate meal frequency.

*Conclusion:* According to the study findings, the proportion of inadequate meal frequency among young children in Ethiopia was higher and also distributed non-randomly across Ethiopian regions. As a result, policymakers and other concerned bodies should prioritize risky areas in designing intervention. Thus, special attention should be given to the Somalia region, the northern part of Amhara, the eastern part of Southern nations and nationalities, and southwestern Oromia.

## Introduction

Minimum meal frequency (MMF) is a percentage of breastfed and non-breastfed infants aged 6 to 23 months who eat nonliquid foods, including milk for non-breastfed infants, the minimum number of times in a day [1]. It shows how frequently children consume meals per day. The minimum number is specific to age and breastfeeding status of the child. Infants and young children are susceptible to malnutrition if they do not receive adequate and frequent meals [2]. The use of poor eating habits and the effects of those practices globally continue to be one of the key barriers to sustained socioeconomic growth and the reduction of poverty [3]. Since children between the ages of 6 and 23 months move from exclusively breastfeeding to semisolid foods or fluids as the main source of nourishment, inadequate meal frequency during this time exposes them to malnutrition [4, 5].

Even yet, the first two years of life are critical for fostering the healthiest growth and behavioral development [6], Inadequate meal utilization and other inappropriate infant and young child feeding (IYCF) practices have long-term consequences such as stunting, delayed cognitive development, and a markedly increased risk of many infectious and chronic illnesses [7, 8]. Despite efforts being made, Ethiopia ranks last among East African nations for newborn and early child feeding practices [9]. A previous study found that children in Ethiopia aged 6–23 months had a minimum meal frequency of 56% [10]. This demonstrates that nearly half of children still consume inadequate meals.

Inappropriate young children feeding practices have a variety of contributing factors that make up their multifaceted cause [11, 12]. In previous studies, household family size, wealth index, birth order, place of residence, age of the child, maternal ANC visit, media exposure, and cultural and traditional views of the community were recognized as factors associated with the attainment of the minimum meal frequency among children [2, 13–15].

Even though Ethiopia has been working in accordance with WHO implementing strategies like the Sustainable Development Goals, which aim to end all forms of malnutrition by 2030, and other Ethiopian government-initiated strategies like the 2008 National Nutrition Program, Seqota Declaration, and Growth and Transformation Plan II [16], which aim to improve the levels of child feeding practices, it is still on the list of nations with low levels of meal frequency and other nutritional indicators [17].

Across the globe, only a small percentage of children receive adequate meals on a regular basis. Inadequate meal frequency habits have a definitive impact on nearly all aspects of children’s development and health [13]. Though there are studies done on meal frequency and determinants in Ethiopia, there is little information on spatial distribution and its determinant factors of inadequate meal frequency at the national level. Therefore, we aimed to investigate the spatial distribution and determinants of inadequate meal frequency among young children in Ethiopia. As minimum meal frequency is an indicator of young infant feeding practice, understanding the significant hot-spot area of inadequate meal frequency helps to evaluate the feeding practice of young children, and the cold-spot area evaluates national progress towards nutritional interventions against under nutrition. Furthermore, the government, policymakers, and other relevant authorities can identify prioritized areas or hot spots for intervention in improving young children’s feeding in Ethiopia.

## Methods

### Study design, setting and periods

The most recent EDHS data (2019) was used to evaluate the breadth and geographical distribution of inadequate meal frequency and associated factors among young children in Ethiopia. The Demographic Health Survey (DHS) is a multi-round cross-country survey that examines population health with a focus on maternal and child health and nutrition with face-to-face interviews of women aged 15 to 49. The Ministry of Health and Ethiopia’s Central Statistical Agency (CSA) worked together to collect the data. A stratified two-stage cluster sampling technique was applied. In the first stage, 305 enumeration areas (EAs) were selected, with 105 probabilities corresponding to the size of the EAs (93 in urban regions and 212 in rural areas). The second stage involved the random selection of 30 households from each cluster. Kids Record (KR) files, which contain information on women and children, were used in this study. Significant variables related to inadequate meal frequency were retrieved from the data set. The study included kids who were between the ages of 6 and 23 months during the time of the survey. Thus, 1,610 individuals made up the study’s overall weighted sample. A multilevel binary logistic regression model was applied. Both crude and adjusted odds ratios with a 95% CI were reported as potential predictors of inadequate meal frequency.

### Study setting

The study was conducted in Ethiopia, a country located in the horn of Africa with a geographical coordinates of 9.145° N latitude and 40.48 97° E longitude [18]. The nation’s overall surface area is estimated to be 1,126,829 km^2^. Its neighbors are Djibouti, Eritrea, Kenya, Somalia, South Sudan, Sudan, and Somaliland. There are twelve administrative regions in Ethiopia—namely Tigray, Afar, Amhara, Gambela, Harari, Oromia, Somali, Southern Nation Nationalities and Peoples Region (SNNPR), Sidama, and the recently added South West Peoples—and two city administrations (Addis Ababa and Dire Dawa). Each of the country’s ten administrative regions and two administrative cities are organized into zones, districts, towns and kebeles (the smallest administrative units) [19]. More than 84% of the population of Ethiopia resides in rural areas. Ethiopia uses a three-tier healthcare delivery system: primary healthcare (PHC) consists of health posts, health centers and primary hospitals; secondary care consists of zonal hospitals; and tertiary care consists of comprehensive specialized hospitals. It has health extension workers, nurses, health officers, midwives, and doctors currently working in the private and government health institutions.

### Sample and populations

The source population was all children aged 6–23 months, whereas the selected children aged 6–23 in the EAs were the study populations. A two-stage stratified cluster sampling technique was employed. Stratification was done by separating each region into urban and rural areas. In stage one, 305 EAs were selected using probability sampling. In the second stage, households were selected systematically in each EA. Then, young infants were selected using KR files, and important variables were selected from the data set.

## Study Variables

### Outcome variable

The inadequate meal frequency indicators utilized were taken from data of EMDHS 2019. We used five data sets (KR) files from EMDHS 2019 to compute inadequate meal frequency and associated variables. Inadequate meal frequency was recorded as “yes” for inadequate meal frequency and “no” for adequate meal frequency. Minimum meal frequency is defined as two feedings of solid, semisolid, or soft foods for breastfed infants aged 6–8 months; three feedings of solid, semisolid, or soft foods for breastfed children aged 9–23 months [20, 21]; and four feedings of solid, semisolid, or soft foods or milk feeds for non-breastfed children aged 6–23 months, whereby at least one of the four feeds must be a solid, semisolid, or soft food [6]. For this study, MMF was categorized into two groups. Thus, children who feed at least the minimum times (i.e., two times for 6–8 months breastfed children, three times for 9–23 months breastfed children, and four times for non-breastfed children) are considered to have adequate minimum meal frequency. On the other hand, youngsters who consume less than the aforementioned minimal number of meals each day are considered to have inadequate minimum meal frequency.

### Independent variables

The independent variables were reviewed from different literatures, and these include factors related to child, maternal and community characteristics that affect children’s nutritional status [17, 22]. The primary exposure factors included household wealth index, place of delivery, mother’s marital status, family size, maternal education, head of household, counseling on breastfeeding, child sex, birth order, child age in months, dietary diversity score, religion, residence, community literacy, and community poverty level. Community-level factors were aggregated from individual-level factors at cluster level.

### Data collection procedure

The DHS Program granted us permission to collect and use the data from http://www.dhsprogram.com for this study after we asked permission. Before releasing the data collected from the DHS to the public, participant identification was erased, and institutional ethical approval was waived to ensure compliance with the rules governing the protection of human beings. ArcGIS coordinates are gathered only for the entire EA and not for specific houses during in-person surveys, and the measured coordinates are dispersed at random over a broad geographic region.

### Data management and analysis

The application of sample weights to account for the uneven likelihood of selection between the strata helped to restore the survey’s representativeness. For nonspatial analysis, we utilized STATA v.14 to weight the survey data and generate descriptive and summary statistics. For spatial analysis we utilized ArcGIS version 10.8 and Sat Scan version 9.6 for the visual presentation of inadequate meal frequency at the regional and district levels. Ethiopia’s district demarcation shape file was downloaded from the database of the Ethiopian Central Statistical Agency (CSA).

### Spatial autocorrelation analysis

The global spatial autocorrelation (Global Moran’s I) statistic measure was used to assess whether the distribution of MMF among children in the research area was dispersed, clustered, or randomly distributed. It was used to find the spatial autocorrelation of the inadequate meal frequency: calculated Moran’s I values close to a value of −1 indicate that children’s inadequate meal frequencies are dispersed, while those close to a value of 1 indicate that children’s inadequate meal frequencies are clustered, and those close to a value of 0 indicate that children’s inadequate meal frequencies are distributed randomly. The existence of spatial autocorrelation is indicated by a statistically significant Moran’s I (p <0.05).

### Hot-spot analysis

Spatial analysis tools, which are final confirmatory spatial studies, were carried out with the aid of Sat Scan and ArcGIS. The Sat Scan pinpoints geographical regions with noticeably higher aggregate rates. In contrast to the distributions outside of the cluster windows, its findings demonstrate hot-spot regions in circular windows, indicating that distributions inside the windows are greater than outside. ArcGIS was used to locate hot spots with a high cluster of inadequate meal frequency and cold spots with low-level clusters. Geti Ord Gi* information for each region in Ethiopia was utilized to conduct a hot-spot analysis of the required minimum meal frequency. Z-score, which measures clustering’s statistical significance, was determined by the p-value. The null hypothesis cannot be rejected if the p-value is larger than 0.05 and the z-score is between 1.96 and +1.96, in which case the pattern shown is most likely the consequence of random spatial processes. If the z-score is outside the range, the observed spatial pattern is likely too unique to be due to chance, and the p-value will be significant. A “hot spot” is indicated by a high Gi* statistical output, whereas a “cold spot” is indicated by a low Gi*.

### Spatial interpolation of minimum meal frequency among young children

The spatial interpolation approach was utilized to predict the unsampled values from the sampled values. To forecast and create uniform surfaces for children’s minimal meal frequency, the kriging interpolation approach was utilized. As a result, in this study, conventional kriging was used to assess the burden of inadequate minimum meal frequency.

### Cluster analysis

Spatial analysis tools Sat Scan and ArcGIS were used to perform final confirmatory spatial studies. The Sat Scan identifies areas geographically where significant higher aggregate rates. Its results show hot-spot areas in circular windows, showing that distributions inside the windows are higher than predicted when compared to the distributions outside of the cluster windows [23]. Hot-spot areas with a high cluster of inadequate meal frequency and cold-spot areas with low-level clusters were identified in ArcGIS.

### Random effect analysis

Random effects, or measures of variation of the outcome variable, were estimated by the median odds ratio (MOR), intra-class correlation coefficient (ICC), and proportional change in variance (PCV). The ICC and PCV were computed to measure the variation between clusters. Taking clusters as a random variable, the ICC reveals the variation of inadequate minimum meal frequency between clusters as ICC = VC/ (VC+3.29) × 100% where VC is variance of cluster. The MOR is the median value of the OR between the area of highest risk and the area of lowest risk for inadequate minimum meal frequency when two clusters are randomly selected, using clusters as a random variable: MOR = _e_^0.95√Vc^_._ where vc is the variance of a cluster Moreover, the PCV demonstrates the variation in the prevalence of inadequate meal frequency explained by factors and computed as PCV = (Vnull-Vc)/Vnull, where Vnull, is the variance of null model & VC variance of cluster. The fixed effects were used to estimate the association between the likelihood of inadequate meal frequency and individual- and community-level independent variables. It was assessed, and the strength was presented using an AOR and 95% CI with a p-value of 0.05. Because of the nested nature of the model, deviation = −2 (log likelihood ratio) was used to compare models, and the model with the lowest deviance was selected as the best-fit model. The variables used in the models were verified for multicollinearity by measuring the variance inflation factors (VIF), with the findings falling within acceptable limits of one to ten.

### Associated factors of minimum meal frequency

A two-level multivariate logistic regression analysis was performed to examine the effects of individual- and community-level characteristics on inadequate meal frequency and to determine the extent to which factors at the individual and community levels explain minimum meal frequency variation in young infants. In the EDHS data, children are nested within a cluster; thereby infants within the same cluster were more similar to one another than were children in different clusters. Therefore this violates the standard regression model assumptions, which are independence of observations and equal variance across the cluster assumptions. This implies the need to take into account between-cluster variables by using an advanced model. Therefore, a multilevel random intercept logistic regression model was fitted to estimate the association between individual-level and community-level factors and the likelihood of inadequate minimum meal frequency. Models were compared based on deviance (-2log likelihood) since the models were nested. Log-likelihood and ICC were computed to measure the variations between clusters. The ICC indicates the degree of heterogeneity of minimum meal frequency between clusters.

### Multilevel logistic regression

A two-level multiple logistic regression model was fitted to investigate factors associated with MMF. In the analysis, four models were fitted. The first (null) model, which contains only the outcome variables, assesses the degree of intra-cluster variation in inadequate meal frequency. The second model contains individual-level variables, the third model contains only community-level variables, and the fourth model contains both individual-level and community-level variables. A p-value of 0.05 was used to define statistical significance. AORs with corresponding 95% CIs were calculated to identify independent predictors of MMF. To evaluate the variation between clusters, the ICC, MOR, and PCV statistics were calculated. Median Odds Ratio is a measure of unexplained cluster heterogeneity, while ICC was employed to explain cluster variation. PCV measures the total variation attributed to individual-level and community-level factors in the multilevel model as compared to the null model PCV. For model comparison, likelihood and deviance were used [24].

### Research ethics approval

The study was based on a secondary analysis of EMDHS data from 2019. The authors requested the measure DHS by briefly stating the objective of this analysis, and access was granted to use the data on the website (http://www.dhsprogram.com).

## Results

### Spatial autocorrelation

**Figure 1:**
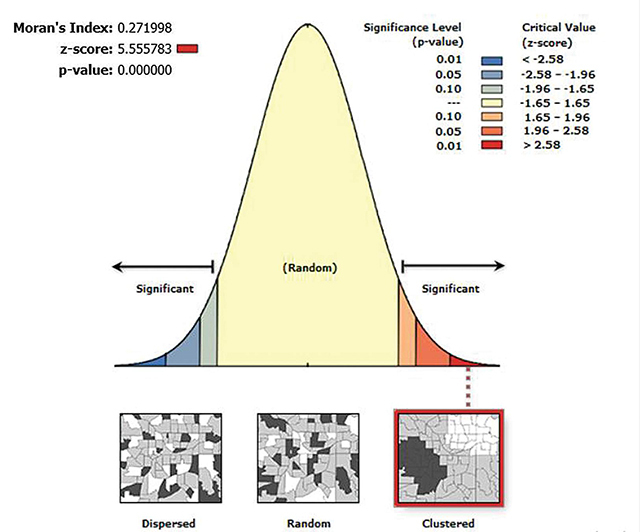
Spatial autocorrelation result of inadequate meal frequency among young children aged 6–23 months in Ethiopia.

The geographical autocorrelation analysis revealed that among Ethiopian children aged 6 to 23 months, there is a significant regional variation in inadequate meal frequency distribution across the region of the country. The Global Moran’s index value, p-value, and z-score values in this study were 0.272, 0.000, and 5.56, respectively. This suggests that Ethiopia’s inadequate minimum meal frequency has significant geographical variations (Figure 1).

### Hot-spot analysis of inadequate minimum meal frequency

**Figure 2:**
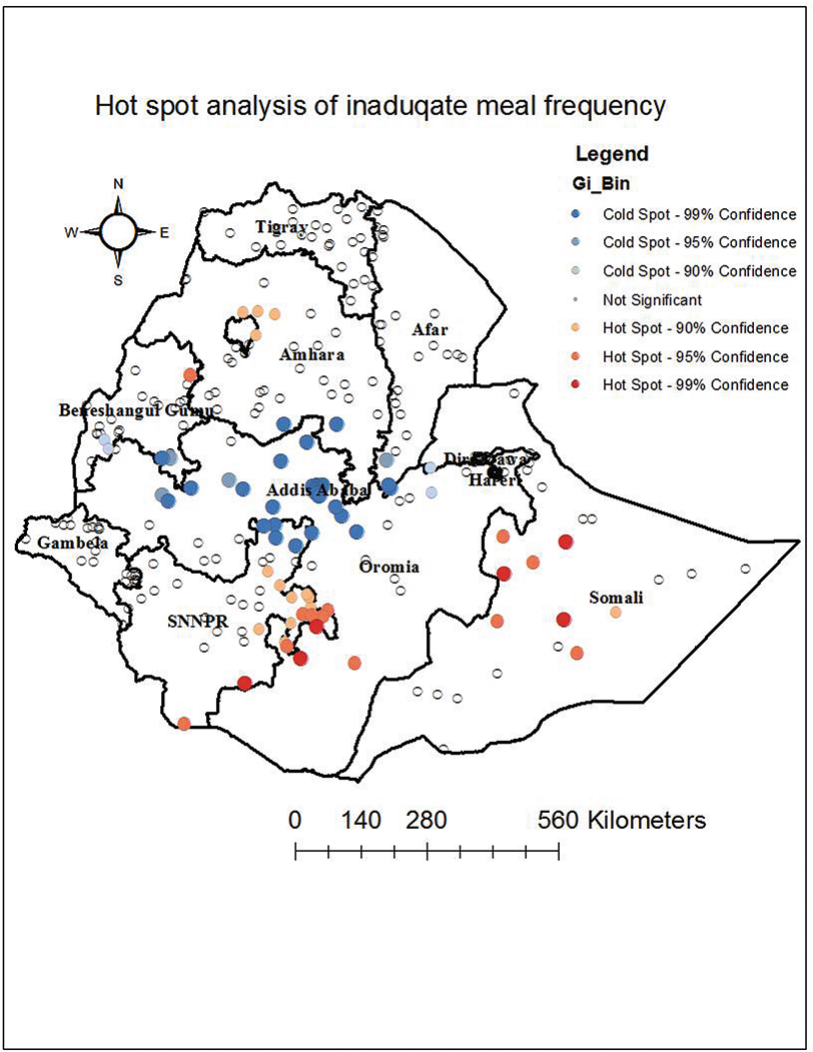
Hot-spot analysis of inadequate meal frequency among children aged 6–23 months in Ethiopia.

This study found a significant clustering of inadequate minimum meal frequency in Somali, South western Oromia, Northern Amhara, and the eastern part of SNNP (Figure 2).

**Figure 3:**
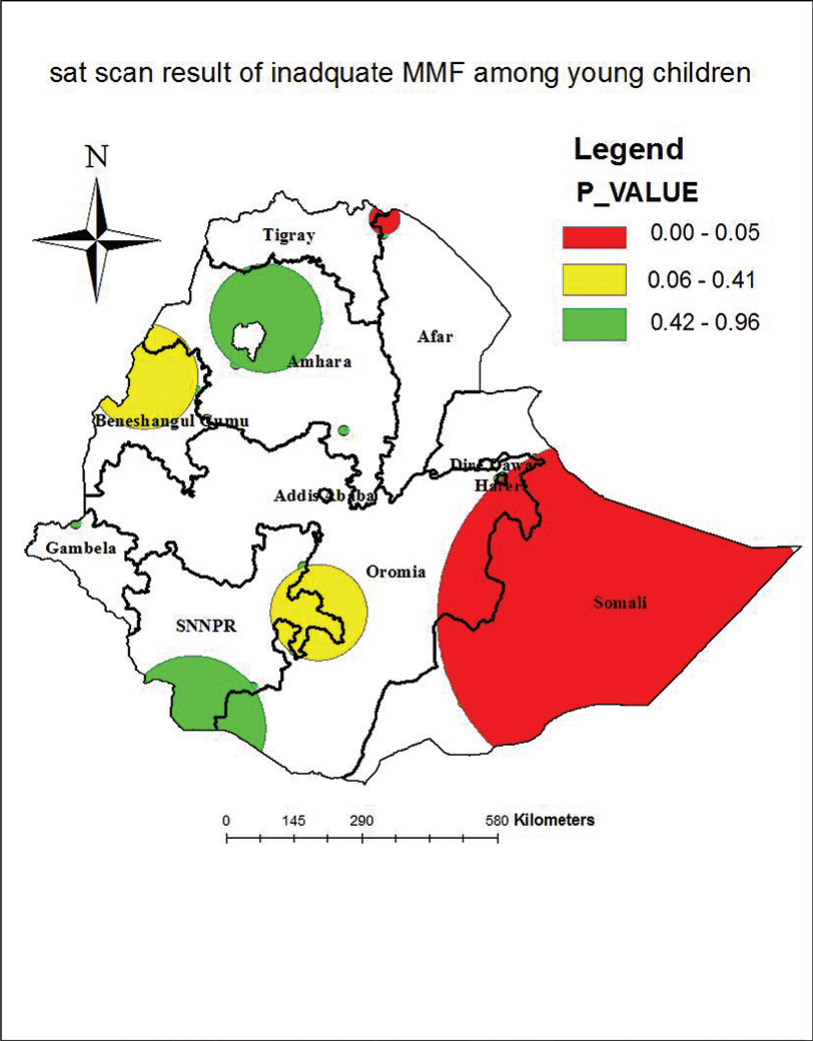
Sat Scan analysis maps of inadequate meal frequency among children aged 6–23 months in Ethiopia.

A total of 44 significant clusters were found in the Sat Scan windows, of which 27 were primary clusters (most likely), 2 were secondary clusters (more likely), and 15 were tertiary clusters. The primary Sat Scan window was detected in almost all parts of the Somali region, on the northern border of Tigray and Afar regions at 6.639662 N and 44.465855 E, with a radius of 390.28 km, with a total population of 170, and 115 cases of inadequate meal frequency with an Relative Risk (RR) of 1.51 and an Log Likelihood Ratio (LLR) of 15.9 (p-value of 0.0001). This shows that children inside this Sat Scan window were 1.51 times more likely to have inadequate minimum meal frequency than children outside of the window. The secondary Sat Scan window was detected in Beneshangul Gumuz, the western part of SNNP, and the eastern parts of Oromia at 14.300432 N, 39.911831 E, a radius of 32.54 km, with a population of 19, and 18 cases of inadequate meal frequency detected with an RR of 2.03 and an LLR of 10.3 (p-value of 0.0087) (Figure 3).

### Spatial interpolation

**Figure 4:**
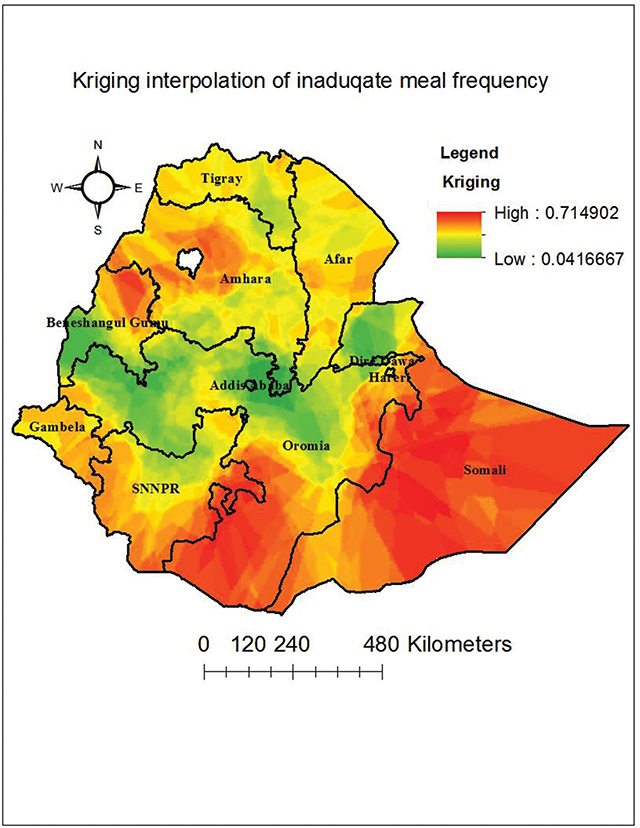
Kriging interpolation map of inadequate meal frequency among children aged 6–23 months in Ethiopia.

**I**nadequate minimum meal frequency in Ethiopia was calculated in an unsampled region using a standard kriging interpolation map. There were found to be high inadequate minimum meal frequencies in Somalia, western Oromia, northern Amhara, and the eastern part of SNNP; whereas low predicted inadequate minimum meal frequency was observed in Beneshangul Gumuz, Addis Ababa, Dire Dawa, Oromia, Eastern Tigray, and the northern parts of Amhara (Figure 4).

### Random effect analysis

The ICC value in the null model indicates 17.29% of the total variation in inadequate minimum meal frequency was due to the difference between clusters. When we randomly selected children from two clusters, the MOR value in the null model, which was 2.19, showed that children from a high-risk cluster had two times greater likelihood to have inadequate minimum meal frequency than children from a low-risk cluster. Furthermore, the final model’s PCV showed that both individual- and community-level variables could account for around 37.8% of the variation in inadequate minimum meal frequency. Model IV had the lowest deviation, making it the best model in terms of model fitness.

## Socio-Demographic Characteristics of Young Infants

A total of 1,610 young infants (825 male and 785 female) participated and were included in the analysis. Almost three-fourths (74.16%) of infants resided in rural areas. Nearly half of mothers (48.7%) had no formal education, and the majority (95%) of mothers were married. More than half (51.24%) of children were male. The majority of children had no prenatal care Post Natal Care (PNC) visit (86.88%). The majority of mothers were married (93.41%). More than three-fourths (78.93%) of young children had inadequate dietary diversity. More than half (56.34%) of children were delivered at a health institution. More than three-fourths (78.08%) of households were headed by a male.

### Prevalence of inadequate minimum meal frequency

The prevalence of inadequate minimum meal frequency was 30.56% (95% CI: 28.33–32.88). The highest prevalence was observed in the Somalia region (50.35%) and the lowest observed in Addis Ababa (15.22%).

### Factors associated with inadequate meal frequency among young infants in ethiopia, EDHS 2019

A multivariable multilevel logistic regression model was fitted to identify factors associated with inadequate meal frequency in Ethiopia. Accordingly, ANC visits during pregnancy, child age, community education, marital status, and wealth index were significantly associated with inadequate meal frequency among children aged 6–23 months in Ethiopia. Inadequate minimum meal frequency among children aged 6–8 were 1.47 times (AOR = 1.47; 95% CI: 1.06–2.02) and 9–11 were 1.48 times (AOR = 1.48; 95% CI: 1.05 −2.08) more likely to have inadequate minimum meal frequency as compared to children aged 12–23 months. Children born from married mothers were 50% less likely to have inadequate minimum meal frequency (AOR = 0.5; 95% CI; 0.28–0.92) as compared to unmarried mothers. Children from mothers having no ANC visit had 1.56 times (AOR = 1.56, 95% CI: 1.07–2.29) and those having only one ANC visit had two times (AOR = 2.32, 95% CI 1.18–4.54) the inadequate minimum meal frequency as compared to children from mothers having at least four ANC visits. Children from uneducated communities were 5.68 times more likely (AOR = 5.68 95% CI: 4.37–7.39) to have inadequate meal frequency as compared to educated communities.

## Discussion

This study found that inadequate meal frequency is not random at the national and regional levels. Significant clusters of inadequate meal frequency were discovered in the Somali region, southwestern Oromia, Northern Amhara, and the eastern part of SNNP. In this study, the proportion of children aged 6–23 months with inadequate minimum meal frequency from a 2019 EMDHS report was 30.56% (95% CI: 28.33–32.88). This is a worrying indicator that shows the issue is still a major nutritional concern that needs to be given the necessary attention by developing all-encompassing comprehensive nutritional solutions. This finding was lower than that of a previous study done in Ethiopia of 56.4% [25]. This might be due to Ethiopia's overall economic improvements and nutritional interventions like adoption of the national nutritional program by the Ethiopian government with other stakeholders, but this finding is higher than the study done in Bangladesh, Indonesia, and India [26–29]. This might be because Bangladesh, India, and Indonesia have more advanced and developed socioeconomic systems than Ethiopia’s, as shown by the countries’ respective gross domestic products: Ethiopia had a GDP of 111.3 billion dollars, while Indonesia had a GDP of 1.186 trillion dollars, Bangladesh had a GDP of 416.3 billion USD and India had a GDP of 3.173 trillion dollars. Thus, having an economic advantage enables them to purchase available nutrients that help their children have a better nutritional status. Additionally, the divergence may be the result of government priorities and variation on child feeding practices among nations.

**Table 1:** Significant Spatial Clusters of Inadequate Meal Frequency among Children in Ethiopian Mini Demography Health Survey, 2019 (n = 1,610).

CLUSTER	ENUMERATION AREAS	COORDINATE (RADIUS)	POPULATION	CASES	RR	LLR	P-VALUE
1	135, 123, 140, 137, 138, 124, 131, 145, 132, 122, 134, 136, 142, 133, 139, 129, 121, 130, 107, 250, 141, 128, 248, 249, 255, 254, 247	(6.639662 N, 44.465855 E) / 390.28 km	170	115	1.51	15.9	0.0001
2	39, 35	(14.300432 N, 39.911831 E) / 32.54 km	19	18	2.03	10.3	0.087
3	181, 186, 182, 185, 187, 183, 117, 113, 184, 115, 178, 116, 172, 188, 203	(6.721681 N, 38.648685 E) / 102.43 km	90	61	1.48	8.12	0.075
4	159, 160, 158, 161, 162	(11.267438 N, 35.292873 E) / 112.65 km	31	24	1.66	6.00	0.4
5	193, 202	(4.495034 N, 36.230625 E) / 155.19 km	17	14	1.75	4.5	0.82
6	67	(10.236394 N, 39.142029 E) / 0 km	6	6	2.2	4.5	0.89
7	218	(8.436117 N, 33.991501 E) / 0 km	10	9	1.91	4.15	0.93
8	82, 84, 83, 57, 56, 59, 74, 54, 81, 58, 78, 75	(12.387114 N, 37.630096 E) / 116.41 km	73	46	1.35	3.8	0.96

The geographical distribution of inadequate MMF varies greatly across Ethiopia, with a Global Moran’s Index of 0.272 at p-value 0.00001 [30–32]. This is in line with a study done in Ethiopia: significant clustering of inadequate meal frequency was observed in Northern Amhara [25], Somali, Southwestern Oromia, and the eastern part of SNNP. This demonstrated that the problem persisted and that neither the government nor any other concerned body had made any significant interventions. Drought is a recurrent occurrence in the eastern portion of Ethiopia, which puts the region first in failing to meet nutritional needs [33].

The random-effects logistic regression model showed that both individual- and community-level factors contributed to the variance in the inadequate minimum meal frequency among children aged 6 to 23 months in Ethiopia. The proportional change in variance for the final complete model (model IV) showed that both individual- and community-level factors accounted for around 17% of the variation observed for inadequate minimum meal frequency of children aged 6 to 23 months.

**Table 2:** Random effect and model fitness for the assessment of inadequate minimum meal frequency among young children in Ethiopia (n = 1,610).

PARAMETER	NULL MODEL	MODEL I	MODEL II	MODEL III
**Variance**	0.69	0.43	0.34	0.43
**ICC**	17.29%	11.55%	9.26%	9.84%
**MOR**	2.20	1.87	1.73	1.86
**PCV**	Reference	37.56%	51.16%	37.8%
**Log likelihood**	–1086.35	–1006.89	–1039.39	1000.41
**Deviance**	2,172.69	2013.77	2.078.78	2000.82

**Table 3:** Socio-demographic characteristics of mother–infant pairs for inadequate meal frequency among young infants aged 6–23 months in Ethiopia, EMDHS 2019 (n = 1,610).

VARIABLE	RESPONSE	FREQUENCY	PERCENT (%)
Child age	6–8 9–11 12–23	285 257 1,066	17.72 15.98 66.29
Child sex	Male Female	807 767	51.27 48.73
Birth order	1 2–3 ≥4	512 623 475	31.80 38.69 29.51
Mother's education	No education Primary Secondary Higher	771 551 144 108	48.98 35.01 9.15 6.86
Maternal age	15–19 20–34 ≥35	118 1,201 255	7.5 76.3 16.2
ANC visit	No Once Twice Three times Four & above	400 57 131 306 641	26.06 3.71 8.53 19.93 41.76
PNC visit	No Yes	1,331 201	86.88 13.12
Marital status	Unmarried Married	106 1502	6.59 93.41
Dietary diversity	Poor Good	1,191 318	78.93 21.07
Wealth index	Poorest Poor Middle Rich Richest	489 262 236 218 403	30.41 16.29 14.68 13.56 25.06
Place of delivery	Home Health institution	703 907	43.66 56.34
No. of household members	≤5 ≥6	789 785	50.13 49.87
Household head sex	Male Female	1,229 345	78.08 21.92
No. of children under age 5	≤2 ≥3	1,358 216	86.28 13.72
Counseling on breastfeeding	No Yes	917 630	59.28 40.72
**Community-level factors**			
Residence	Urban Rural	25.6 1,171	25.6 74.4
Community poverty level	Low High	737 837	46.82 53.18
Community illiteracy level	Low High	854 720	54.26 45.74
Region	Tigray Afar Amhara Oromia Somali Benishangul SNNPR Gambela Harari Addis Ababa Dire Dawa	128 166 162 189 143 145 180 122 122 92 125	21.09 44.58 23.46 24.87 50.35 24.14 40.00 28.69 32.79 15.22 21.60

**Table 4:** Multivariable multilevel logistic regression analysis result of both individual- and community-level factors associated with minimum meal frequency among young infants in Ethiopia.

INDIVIDUAL- AND COMMUNITY- LEVEL FACTORS		MODEL I AOR (95% CL)	MODEL II AOR (95% CL)	MODEL III AOR (95% CL)
Child age	6–8 9–11 12–23	1.5 (1.09–2.04)* 1.42 (1.02–1.98)* 1		1.47 (1.06–2.02)* 1.48 (1.05–2.08)* 1
Child sex	Male Female	1 0.88 (0.7–1.01)		1 0.89 (0.71–1.02)
Birth order	1 2–3 ≥4	0.9 (0.63–1.27) 0.89 (0.67–1.18) 1		0.88(0.61–1.26) 0.85(0.64–1.15) 1
Mother's education	No education Primary Secondary Higher	1 0.75 (0.56–1.00) 0.7 (0.43–1.14) 0.54 (0.29–0.99)*		1 1.65(0.88–2.5) 1.22(0.67–2.2) 1.24(0.63–2.4)
Maternal age	15–19 20–34 ≥35	0.91 (0.85–1.32) 1 0.92 (0.87–1.34)		0.90 (0.83–1.31) 1 0.93 (0.89–1.37)
ANC visit	No Once Twice Three times Four & above	1.89 (1.32–2.7)* 2.13 (1.12–4.08)* 1.09 (0.69–1.7) 1.47 (1.00–2.04) 1		1.56(1.07–2.29)* 2.32(1.18–4.54)* 0.96(0.59–1.55) 1.34(0.96–1.89) 1
PNC visit	No Yes	1 0.92 (0.63–1.34)		1 0.95(0.64–1.41)
Marital status	Unmarried Married	1 0.56 (0.32–1.00)		1 0.5(0.28–0.92)*
Dietary diversity	Poor Good	1 0.84 (0.57–1.23)		1 0.99 (0.66–1.49)
Wealth index	Poorest Poor Middle Rich Richest	1 0.7 (0.48–1.03) 0.66 (0.44–0.99)* 0.52 (0.34–0.80)* 0.52 (0.34–0.79)*		1 0.76 (0.44–1.31) 0.65 (0.38–1.03) 0.51 (0.32–0.78)^*^ 0.50 (0.30–0.75)^*^
Place of delivery	Home Health institution	1 1.06 (0.78–1.44)		1 0.75 (0.55–1.01)
Number of household members	≤5 ≥5	1 0.95 (0.90–1.01)		1 0.96 (0.92–1.21)
Household head sex	Male Female	1 0.89 (0.85–1.88)		1 0.88 (0.85–2.00)
Number of children under age 5	≤2 ≥3	1 0.86 (0.81–1.84)		1 0.85 (0.81–1.83)
Counseling on breastfeeding	No Yes	1 0.79 (0.59–1.05)		1 0.75 (0.55–1.01)
**Community-level factors**				
Region	Tigray Afar Amhara Oromia Somali Benshagul SNNPR Gambela Harari Dire Dewa Addis Ababa		1.41 (0.7–2.760) 1.55 (0.81–2.98) 1.31 (0.68–2.54) 1.32 (0.69–2.52) 2.00 (1.01–3.97) 1.57 (0.83–3.07) 1.79 (0.93–3.43) 1.19 (0.60–2.36) 1.40 (0.73–2.70) 1.15 (0.59–2.26) 1	1.45 (0.68–3.11) 1.65 (0.49–2.24) 1.28 (0.60–2.72) 1.23 (0.58–2.62) 1.38 (0.61–3.08) 1.28 (0.58–2.82) 1.62 (0.75–3.47) 1.17 (0.52–2.62) 1.10 (0.52–2.33) 0.8 (0.35–1.80) 1
Residence	Urban Rural		0.9 (0.66–1.26) 1	1.07 (0.69–1.68) 1
Community poverty level	Low High		1 1.18 (0.9–1.54)	1 0.83 (0.57–1.22)
Community illiteracy level	Low High		1 5.5 (4.33–6.96)*	1 5.68 (4.37–7.39)*

In our multilevel analysis of EMDHS, we identified major determinants of minimum meal frequency among young infants in Ethiopia. In the multivariable logistic regression model, maternal marital status, ANC visits, family wealth status, child age, and community literacy level were significantly associated with inadequate minimum meal frequency. The odds of getting inadequate meal frequency among young infants from uneducated communities were higher than those from educated communities [34]. This could be because educated people are more likely to comprehend educational nutrition messages, to engage in paid jobs, and to have greater socioeconomic status, all of which could have a good effect on baby-feeding habits [35, 36]. In addition it may also be due to the fact that trained women were more likely to have the necessary information, comprehend the practice of child feeding, and get instruction in child feeding in school that would increase their understanding of the importance of child feeding [37].

Child age was significantly associated with minimum meal frequency in that the odds of minimum meal frequency were lesser among infants aged 6–11 months than those infants aged 12–23 months [1, 17, 38, 39]. This indicates that minimum meal frequency is positively associated with the age of young infants, implying that the practice of minimum meal frequency increases as the child age grows; however, being of a young age increases the odds of inadequate meal frequency. The possible explanation could be brought on by how the mother perceives the younger child and how poorly the infant’s intestines can absorb and digest food. Mothers may also believe that introducing children to bulk meals may increase their risk of developing bowel illnesses. On the contrary, as the child grows older, the frequency of meals rises, the diet becomes more diverse and the child probably gets adequate meals on a regular basis.

The number of ANC visits that the mother attended showed statistical significance with meal frequency; thereby the odds of receiving inadequate meal frequency among infants born to mothers who had no or only one ANC visit during pregnancy were higher when compared to those who had four and above ANC visits [32, 35, 38]. This could be due to ANC service that improves maternal counseling and community conversations program about child-feeding practices, which enhance the understanding of mothers about how to prepare food and feed their children [17].

Maternal marital status was also significantly associated with inadequate meal frequency among young infants in which unmarried women’s children received higher inadequate meal frequency than infants of married mothers [40–42]. Worldwide, husband/partner and family (e.g., grandmother) support for breastfeeding is essential for appropriate IYCF practices in the household [43]. This could be because living with a partner or being married increases family income, which encourages family members to buy and cook more nutritious meals.

Family wealth status was also one factor which significantly associated with inadequate meal frequency, by which households having medium and rich wealth status had better-met nutritional requirements [1]. This could be due to larger home income, which usually means more purchasing power, access to a wider choice of foods, and more money available for expenditure on daycare and nutrition.

## Conclusion

The results of this study indicate that the minimal meal frequency for children, as determined by the WHO dietary assessment, is inadequate. In this survey, the location of inadequate minimum meal frequency was not distributed at random. All parts of the Somali region, the northern part of the Amhara region, the southwestern parts of the Oromia region, and SNNP are hot-spot areas in which inadequate meal frequency still persists. As a result, policymakers and other concerned bodies should prioritize risky areas in designing intervention. Thus, special attention should be given to the Somalia region, the northern part of Amhara, the eastern part of Southern nations and nationalities, and southwestern Oromia.

### Strength and limitation

Since it is based on data from a nationwide survey, the study has the potential to help programmers and policymakers to develop effective intervention at the national level. However, the DHS is primarily dependent on respondents’ self-report, thus there is a chance of recall bias in this study.

## List of Abbreviations

**Table:** 

ANC	Antenatal care
CI	confidence interval
CSA	Central Statistical Agency
EA	enumeration area
EDHS	Ethiopian Demographic Health Survey
GDP	Gross Domestic Product
ICC	intra-class correlation coefficient
IYCF	infant and young children feeding
KR	kids record
LR	logistic regression
MOR	median odds ratio
MMF	minimum meal frequency
PNC	prenatal care
PHC	primary healthcare
PCV	proportional change in variance

## Declaration

### Ethics approval and consent to participate

The data used in our study was obtained from the Measure Demographic Health Survey program website (http://www.dhsprogram.com), through formal request after registering on the website. Then the data will be available for download within two to three days after registering and requesting the data. The website also granted a formal permission letter. The data was anonymized before it was included in the analysis. All the methods were carried out in accordance with relevant guidelines and regulations.

## Data Availability

The datasets used and/or analyzed during the current study are publicly available on (http://dhsprogram.com) website.
